# Photoregeneration of Trimethylsilyl Cellulose as a Tool for Microstructuring Ultrathin Cellulose Supports

**DOI:** 10.3390/molecules191016266

**Published:** 2014-10-10

**Authors:** Archim Wolfberger, Rupert Kargl, Thomas Griesser, Stefan Spirk

**Affiliations:** 1 Chair of Chemistry of Polymeric Materials, University of Leoben, Otto Glöckel-Straße 2, 8700 Leoben, Austria; 2 Institute for the Engineering and Design of Materials, University of Maribor, Smetanova Ulica 17, 2000 Maribor, Slovenia; 3 Institute for Chemistry and Technology of Materials, Graz University of Technology, Stremayrgasse 9, 8010 Graz, Austria

**Keywords:** cellulose thin films, cellulose regeneration, patterning of cellulose, photoregeneration

## Abstract

Microstructured thin films based on cellulose, the most abundant biopolymer on Earth, have been obtained by UV-irradiation of acid-labile trimethylsilyl cellulose thin films in the presence of *N*-hydroxynaphtalimide triflate as photoacid generator. We demonstrate that this photoregeneration process can be exploited for the manufacture of cellulose patterns having feature sizes down to 1 μm, with potential applications in life sciences.

## 1. Introduction

**Scheme 1:**
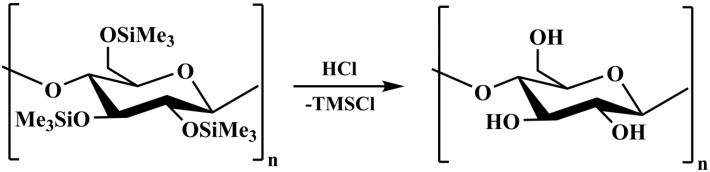
Acid vapor phase regeneration of TMSC to cellulose. Note that the actual DS_Si_ of the TMSC used was 2.8.

Cellulose is the most abundant biopolymer on Earth, offering a variety of properties that can be hardly found in any other single material [1]. As a consequence, its applications are innumerable and cover mass use products such as papers, fibers, textiles, packaging materials, and hygienic products, as well as support materials in chromatography, life sciences and materials for medicine to mention the most important ones [1]. Despite the fact that cellulose is a very versatile base material, one of its major drawbacks is its inherent insolubility in common organic solvents and water. As a result, the processing and shaping of cellulose can be challenging and potentially limiting to its applications to some extent. There are different strategies to overcome this problem, namely the use of so-called cellulose solvents (e.g., NMMO) that allow the dissolution of cellulose or, alternatively, the preparation of organosoluble cellulose derivatives [2]. In both cases, the cellulose (derivative) solution is subjected to regeneration after shaping, *i.e.*, precipitation into a bath where it is insoluble or converted back into cellulose. One of the oldest examples, the Viscose process, is still in industrial use with only minor modifications since its discovery and implementation on an industrial scale at the beginning of the 20th century [3]. The main idea of this process is the conversion of rather insoluble cellulose into a soluble xanthogenate, which is converted back to cellulose after the shaping procedure(s) (e.g., spinning). Later, this concept was adapted by Lenzing AG and led to the development of the so called silyl technology. For this purpose, the cellulose is subjected to *O*-silylation, resulting in the formation of cellulose silyl ethers (e.g., trimethylsilyl cellulose, TMSC) which can easily be cleaved by acidic treatment (e.g., dilute H_2_SO_4_) to give cellulose [4]. Although a complete life cycle assessment has been demonstrated in the early 1980s, this technology has not been applied so far by any suppliers for the production of cellulosic materials, although resulting fiber and film properties have been described as promising [4,5]. In the beginning of the 1990s, TMSC was rediscovered by the Klemm group who opened a new chapter in the preparation of cellulosic materials by introduction of TMSC as precursor for the preparation of ultrathin cellulose films by exposure to acidic HCl vapors (“vapor phase regeneration”, Scheme 1) [6].

This concept has been adapted by Kontturi *et al.* who introduced the spin-coating procedure for the preparation of such films allowing for a fast and highly reproducible manufacturing process with tailored film properties [7]. Since these films are highly amorphous [8,9], they have been used as model systems to study the interaction capacity of different kinds of biopolymers (e.g., other polysaccharides, proteins, *etc*.) with amorphous cellulose, very often with the final aim of simulating adsorption processes on “bulk” materials such as papers and fibers [10,11,12]. In some other studies, the aim was to make use of the intrinsic properties of the cellulosic support in order to generate functional materials for different purposes (e.g., for immobilization of BSA [13,14,15,16], antibodies [13,14,17], DNA [18], as model for fiber-fiber bonds in paper [19], or as dielectric material in organic thin film transistors [20]). Our group has been extensively working on the preparation of patterned cellulose thin films using either macrosized masks [21,22] or PDMS molds in combination with soft lithography and enzymes [23] to realize macro- and microstructured ultrathin cellulose supports for different applications. However, while these procedures are reproducible they are less suited for broader application since they are too laborious and time consuming. As a consequence, a large scale production of these materials is *per se* unlikely, limiting the applicability of the already described approaches. The idea of this communication is to introduce the use of light to regenerate cellulose (“photoregeneration”) at the example of ultrathin cellulose supports. For this purpose, we apply a concept from semiconductor industry where photoacid generators (PAG) are widely used to alter materials’ properties.

## 2. Results and Discussion

PAGs generate an acid upon exposure to UV light and the formed acid induces either crosslinking or cleaves acid labile bonds in a polymer resulting in a different solubility behavior of the polymer. For this purpose, we added 2 wt % of *N*-hydroxynaphtalimide triflate (NHT) to TMSC dissolved in chloroform (1 wt %). In order to fabricate thin films, these solutions were deposited onto a doped silicon wafer and subjected to spin coating (v = 2000 rpm, a = 1000 rpm·s^−1^, t = 60 s) according to published literature procedures.

**Figure 1:**
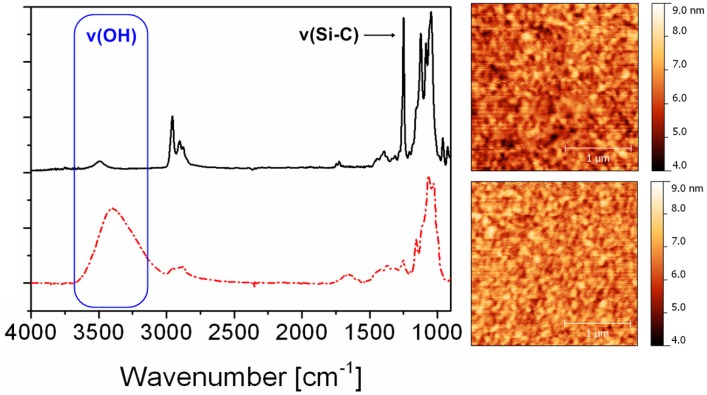
Comparison of IR spectra (transmission) and AFM images (scan size 2.5 × 2.5 μm^2^) before (upper row) and after photoregeneration of TMSC thin films containing 2 wt % of NHT.

These films (with a layer thickness of approximately 60 nm) have been further illuminated by UV light (λ > 365 nm) for a period of 10 minutes under ambient atmosphere. Upon illumination with UV light, the NHT creates triflic acid regenerating cellulose from TMSC by nucleophilic attack of the trimethylsilyl group. This conversion can be monitored nicely via the corresponding IR spectra (transmission) which clearly show a decrease in intensity of the Si–O–C band (1250 cm^−1^) concomitant with the appearance of the OH valence vibrations at 3400 cm^−1^ (Figure 1).

Surprisingly, the presence of PAGs as well as the photoregeneration procedure do not influence the surface morphology of the films. Both surfaces, the TMSC as well as the photoregenerated cellulose surface do not exhibit any specific surface features and nearly have the same rms roughness. A further proof for the photoinduced conversion of TMSC to cellulose is the wettability with water, which increases after illumination (compare static contact angle of TMSC: 99 ± 1° and photoregenerated cellulose: 27 ± 1°). Since the photoregeneration of TMSC was promising, we became interested whether it is possible to create patterned structures using photolithographic techniques.

**Figure 2:**
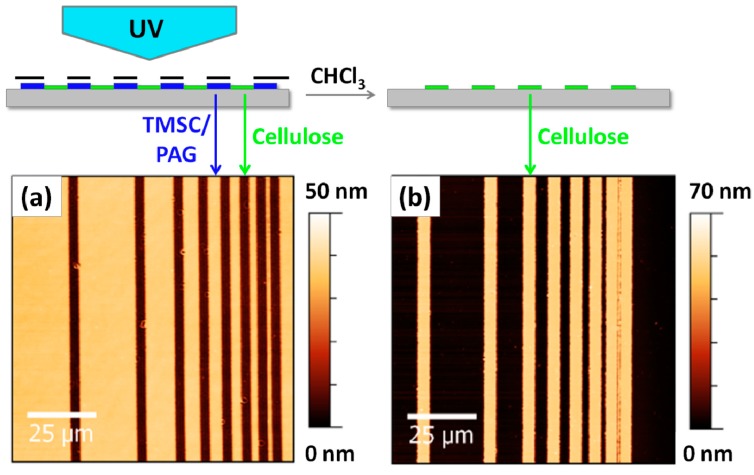
AFM images of TMSC ultrathin films using a photolithographic mask after irradiation (**a**) and rinsing (**b**). The smallest structure has a size of *ca.* 1 μm and wavelengths below 365 nm have been excluded by an emissive filter to prevent degradation of cellulose during the illumination step.

Since photolithography is a common technique to create sub-micron structures by employing masks, the next step was to use a mask aligner. TMSC films are placed on the mask aligner and a mask is placed on top of the films followed by UV illumination. The areas of the TMSC films that are illuminated are converted to cellulose while those that are not remain unaffected by the whole procedure. These remaining TMSC features can be rinsed away using an appropriate solvent (in this case: CHCl_3_), while the cellulose stripes remain on the silicon wafer, resulting in a negative type patterned cellulose ultrathin film as shown in Figure 2.

As already reported many times in the literature, the conversion of TMSC thin films to cellulose leads to a reduction in film thickness due to the cleavage of the silyl groups concomitant with the formation of hydrogen bonds in the cellulose and an increase in the density of the film [24,25,26]. Therefore, even directly after illumination a step height is already visible in the AFM images, which originates from the shrinkage of the films induced by photoregeneration. However, after rinsing with organic solvents to remove the remaining TMSC, the step height refers to the layer thickness of the cellulose thin film on the silicon wafer (*ca.* 70 nm for the film depicted in Figure 2).

**Figure 3:**
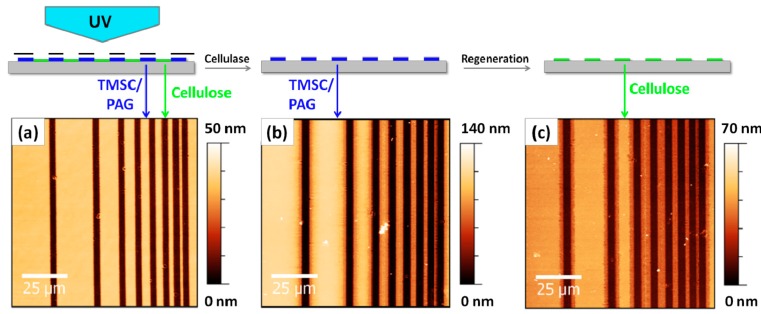
AFM images of TMSC thin films after illumination (**a**), enzymatic treatment (**b**) and regeneration (**c**).

This procedure is not limited to ultrathin films (layer thickness below 100 nm) but can be also applied to thin films (definition: layer thickness in the low micrometer regime). Additionally, it is also possible to employ enzymes for alternative patterning steps. The fabrication of positive type patterns can be realized by using cellulose-digesting enzymes *i.e.*, cellulases, which are deposited directly after the illumination step onto the films. While the photoregenerated cellulose areas on the thin films are readily digested, the TMSC features remain untouched. A further illumination of the remaining TMSC film yields positive type patterned photoregenerated cellulose thin films again. An example is shown in Figure 3, where a TMSC film with an original layer thickness of *ca.* 140 nm is subjected to UV illumination followed by enzymatic treatment using cellulases (18 h, 37 °C, pH 4.8).

After an additional regeneration step, the positive type patterned cellulose structure is obtained, which is accompanied again by a decrease in layer thickness. It can be clearly seen that even for films with an original layer thickness above 100 nm the patterning procedure is rather effective. However, when using enzymes in combination with a larger layer thickness (>100 nm) the resulting features are not as defined as for the negative type patterned films, which can be also related to the long exposure time of the surfaces against the aqueous enzyme solution (18 h). While in principle negative type patterns can be obtained in a destructive way resulting in oxidized products at the edges of the features as well (e.g., using laser lithography [27]), this photo patterning procedure allows for the creation of positive and negative type patterned cellulose films via non-destructive photolithography. Potential byproducts (e.g., PAG) still present in the films can be easily removed by simple rinsing steps using chloroform.

## 3. Experimental

### 3.1. Materials

Unless otherwise stated, all chemicals were obtained from commercial sources and were used without further purification. Trimethylsilyl cellulose with a degree of substitution of DS_Si_ = 2.8 was purchased from the Thuringian Institute of Textile and Plastics Research (Rudolstadt, Germany). *N‑*Hydroxynaphthalimide triflate (electronic grade, ≥99%) and cellulase from *Trichoderma viride* were obtained from Sigma Aldrich (Steinheim, Germany). Silicon wafers were obtained from Taisil Electronic Materials Corp. (Hsinchu, Taiwan) and were rinsed with acetone and cleaned with a polymer cleaning solution (First Contact, Photonic Cleaning Technology LLC, Platteville, WI, USA) after cutting.

### 3.2. Sample Preparation

TMSC films were fabricated by spin coating from 1 wt % chloroform solutions containing 2 wt % of *N*-Hydroxynaphtalimide triflate on silicon wafers.

### 3.3. Photoregeneration

Photoregeneration was carried out with a medium pressure *Hg*-lamp (66990, 100 W, from Newport, Bozeman, MT, USA) equipped with a filter transmissive for wavelengths in the range of 350–450 nm. The light intensity (power density) at the sample surface was measured with a UV radiometer (UV Power Puck, EIT, Inc., Sterling, VA, USA) and was determined as 7.6 mW·cm^−2^ in the spectral range from 250–390 nm (UV-A, UV-B and UV-C).

### 3.4. FTIR Spectroscopy

FTIR spectra were recorded on a Perkin Elmer Spectrum One (Waltham, MA, USA) instrument (spectral range of 850 to 4000 cm^−1^, resolution of 1 cm^−1^) in transmission mode.

### 3.5. Atomic Force Microscopy

AFM micrographs were recorded with a Nanosurf FlexAFM (Langen, Germany) instrument, using silicon AFM probes with a resonance frequency of 190 kHz and a force constant of 48 N·m^−1^ (Tap190AL-G, Budgetsensors, Sofia, Bulgaria).

### 3.6. Photopatterning

Photolithographic patterning was carried out with a mask aligner (500 W HgXe, MJB4, SUSS, Garching, Germany) equipped with a filter transmissive for wavelengths in the range of 365 nm with a measured power density of 9.0 mW·cm^−2^. After photolithographic patterning, a development was performed in chloroform for 10 min at room temperature or via enzymatic digestion using cellulase from *Trichoderma viride* (1 mg·mL^−1^, dissolved in a 100 mM sodium acetate/acetic acid buffer at pH 4.8). The illuminated samples were immersed in 3 to 5 mL of cellulase solution at 37 °C overnight (18 h) and rinsed with buffer and MQ water before AFM imaging.

## 4. Conclusions

In this communication, a new regeneration procedure for cellulose, namely photoregeneration of TMSC, has been described using the example of ultrathin cellulose films. The use of acid-labile cellulose derivatives in combination with PAGs provides a tool to perform photoinduced desilylation reactions on cellulose that can be further exploited to generate patterned cellulose structures, also in combination with enzymes. The smallest feature sizes presented here are in the range of 1 μm, however, we already succeeded to push the limits of the method significantly into the submicron range using two-photon absorption lithography (data not shown). One of the major advantages of this approach is that the process is easy to perform and with some restrictions it is applicable to other polysaccharides (e.g., chitins, chitosans) as well, allowing for new perspectives in the generation of new functional polysaccharide materials.
